# First Report of a Fatal Case Associated with EV-D68 Infection in Hong Kong and Emergence of an Interclade Recombinant in China Revealed by Genome Analysis

**DOI:** 10.3390/ijms18051065

**Published:** 2017-05-16

**Authors:** Cyril C. Y. Yip, Janice Y. C. Lo, Siddharth Sridhar, David C. Lung, Shik Luk, Kwok-Hung Chan, Jasper F. W. Chan, Vincent C. C. Cheng, Patrick C. Y. Woo, Kwok-Yung Yuen, Susanna K. P. Lau

**Affiliations:** 1 Department of Microbiology, Li Ka Shing Faculty of Medicine, The University of Hong Kong, Hong Kong, China; yipcyril@hku.hk (C.C.Y.Y.); sid8998@hku.hk (S.S.); chankh2@hku.hk (K.-H.C.); jfwchan@hku.hk (J.F.W.C.); vcccheng@hkucc.hku.hk (V.C.C.C.); pcywoo@hkucc.hku.hk (P.C.Y.W.); 2 Centre for Health Protection, Department of Health, Hong Kong, China; janicelo@dh.gov.hk; 3 Department of Pathology, Tuen Mun Hospital, Hong Kong, China; h9910303@yahoo.com.hk; 4 Department of Pathology, Princess Margaret Hospital, Hong Kong, China; kristineluk@yahoo.com.hk; 5 State Key Laboratory of Emerging Infectious Diseases, The University of Hong Kong, Hong Kong, China; 6 Collaborative Innovation Center for Diagnosis and Treatment of Infectious Diseases, The University of Hong Kong, Hong Kong, China; 7 Carol Yu Centre for Infection, The University of Hong Kong, Hong Kong, China; 8 Research Centre of Infection and Immunology, The University of Hong Kong, Hong Kong, China

**Keywords:** enterovirus D68, clade, recombination

## Abstract

A fatal case associated with enterovirus D68 (EV-D68) infection affecting a 10-year-old boy was reported in Hong Kong in 2014. To examine if a new strain has emerged in Hong Kong, we sequenced the partial genome of the EV-D68 strain identified from the fatal case and the complete VP1, and partial 5′UTR and 2C sequences of nine additional EV-D68 strains isolated from patients in Hong Kong. Sequence analysis indicated that a cluster of strains including the previously recognized A2 strains should belong to a separate clade, clade D, which is further divided into subclades D1 and D2. Among the 10 EV-D68 strains, 7 (including the fatal case) belonged to the previously described, newly emerged subclade B3, 2 belonged to subclade B1, and 1 belonged to subclade D1. Three EV-D68 strains, each from subclades B1, B3, and D1, were selected for complete genome sequencing and recombination analysis. While no evidence of recombination was noted among local strains, interclade recombination was identified in subclade D2 strains detected in mainland China in 2008 with VP2 acquired from clade A. This study supports the reclassification of subclade A2 into clade D1, and demonstrates interclade recombination between clades A and D2 in EV-D68 strains from China.

## 1. Introduction

Enterovirus D68 (EV-D68) belongs to enterovirus species D (EV-D) in the genus *Enterovirus* of the family *Picornaviridae*. EV-D68 was first isolated from hospitalized children with lower respiratory illnesses in California in 1962 [1], and human rhinovirus 87 was later confirmed as an isolate of EV-D68 based on antigenic and genetic characterization and acid lability testing [2]. Sporadic cases of EV-D68 infection have been reported since 1970 [3], but there has been a significant increase in the number of reports of EV-D68 circulating globally in recent times [4,5,6,7,8,9,10,11]. In 2014, a nationwide outbreak of EV-D68 associated with severe respiratory disease in children and acute flaccid myelitis occurred in the United States (US), affecting over 1100 people in 49 states, 14 of whom died [12]. Most of the EV-D68 strains detected in the US outbreak belonged to clade B1, especially those sampled from acute flaccid myelitis (AFM) cases [13]. In our previous study, we examined the clinical and molecular epidemiology of EV-D68 among hospitalized patients with respiratory tract infections in Hong Kong [14]. Phylogenetic analysis of partial VP1, 2C, and 3D regions of EV-D68 strains in Hong Kong revealed four distinct lineages, clade A1, A2, B1, and the new clade, B3, with the elderly having severe lower respiratory illness exclusively caused by clade A2 [14]. Moreover, strains closely related to the US 2014 outbreak B1 strains were detected as early as 2011 in Hong Kong. These findings suggested that EV-D68 has been evolving into new lineages that might be emerging.

Mutations and recombinations are well-known phenomena in driving enterovirus evolution [15,16,17,18]. However, to date, only one study has demonstrated an intraclade recombination event within an EV-D68 strain US/KY/14-18951, which has arisen from recombination between subclades B1 and B2 [19]. In 2014, there was a fatal case associated with EV-D68 infection in a 10-year old boy in Hong Kong. To determine if a new strain has emerged in Hong Kong and if recombination events have played a role in the evolution of the emergence of new EV-D68 strains in Hong Kong, we sequenced the partial genome of the EV-D68 strain detected from the fatal case. We also randomly selected nine additional EV-D68 strains isolated from patients in Hong Kong during 2012 to 2014 and sequenced their complete VP1, partial 5′ untranslated region (5′UTR), and 2C region. We also determined the complete genome sequences of three EV-D68 strains belonging to three different clades, B1, B3, and D1.

## 2. Results

### 2.1. Clinical Characteristics of Patients with Enterovirus D68 (EV-D68) Infections

The fatal case occurred in a 10-year-old boy (case 1) with an α-thalassemia trait who was residing in mainland China and presented with acute encephalitis, neurogenic pulmonary edema with respiratory failure, autonomic dysfunction, and cardiac arrest requiring intensive care in August 2014 (Table 1). Due to the critical condition and coagulopathy state, lumbar puncture could not be performed. Therefore, he was started on broad-spectrum antimicrobial coverage for possible meningoencephalitis while awaiting investigation results. His condition was complicated by severe hypoxic ischemic encephalopathy and he died nine days after admission despite use of broad-spectrum antimicrobials including cefotaxime, tazobactam-piperacillin, vancomycin, levofloxacin, and acyclovir. *Mycoplasma pneumoniae* and EV-D68 nucleic acids were detected by PCR in his nasopharyngeal aspirate (NPA). The *M. pneumoniae* showed an A2063G mutation indicating macrolide resistance [20]. His peri-mortem cerebrospinal fluid (CSF) obtained after developing confusion and respiratory deterioration was negative for EV-D68 RNA.

Nine other EV-D68 strains were isolated from the nasopharyngeal swabs (NPSs) or NPAs, obtained as part of the diagnostic workup of hospitalized patients from seven regional hospitals in Hong Kong from 2012 to 2014. Including the fatal case, most patients with EV-D68 infections were young children, while one was an elderly female patient (median age 4.5 years, range 2–94 years). Six were male and four were female. The clinical characteristics of the 10 patients with EV-D68 infections are summarized in Table 1. The 94-year-old woman with underlying medical illnesses presented with pneumonia. Six of the young children presented with upper respiratory tract infections (URTI), among which four were complicated by asthmatic exacerbation and one was complicated by Henoch Schonlein purpura. Two other children presented with wheezy bronchitis and febrile wheeze, respectively. Except for one fatal case, the other nine patients survived.

### 2.2. Complete VP1 Gene Sequence Analysis

**Figure 1:**
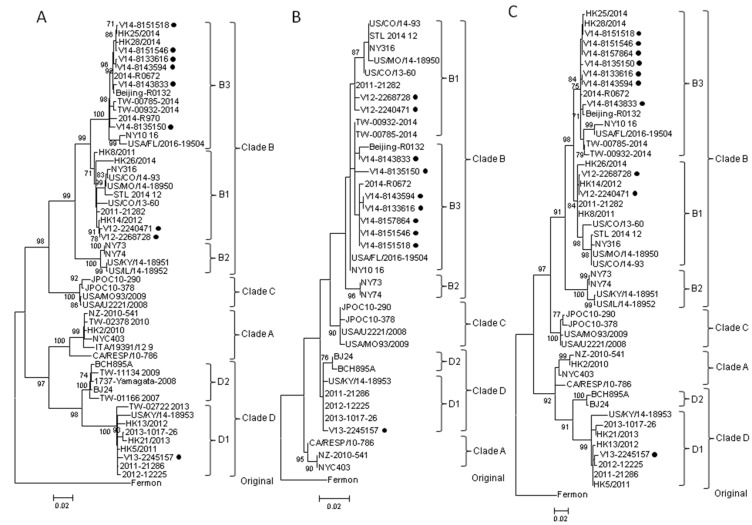
Phylogenetic analysis using VP1, partial 5′UTR and 2C gene sequences of enterovirus D68 (EV-D68) strains.The trees were inferred from (**A**) VP1, (**B**) partial 5′UTR and (**C**) partial 2C sequence data by the maximum likelihood (ML) method, with bootstrap values calculated from 1000 trees. Sequences for 918 nucleotide positions in each VP1 region, 356 nucleotide positions in each 5′UTR region, and 531 nucleotide positions in each 2C region were included in the analysis. Bootstrap values expressed as a percentage are shown at the nodes and the scale reflects the number of nucleotide substitutions per site along the branches. Only bootstrap values >70% are shown. Black circles indicate EV-D68 strains collected for the present study.

Since the strain V14-8157864 from the fatal case could not be grown in cell culture, only partial genomic sequences could be obtained and the complete VP1 sequence was not available. On the other hand, the complete VP1 sequences of the other nine EV-D68 virus isolates were successfully obtained. Phylogenetic analysis showed that they fell into three different subclades of EV-D68. Eight EV-D68 strains were clustered with other EV-D68 strains of clade B (Figure 1A). Among these eight strains, two strains from the year 2012 belonged to subclade B1 (nucleotide divergence of 0.2–3.5% for strains within B1), while six strains from the year 2014 belonged to subclade B3 (nucleotide divergence of 0.1–2.7% for strains within B3). The nucleotide divergence of 2.6–5.3% was observed between subclade B1 and subclade B3 strains (Table 2).

One EV-D68 strain from the only elderly case (case 4) in this study was most closely related to subclade A2. However, recent studies demonstrated that the previously recognized A2 strains should be classified as clade D [21,22]. This was further supported by our in-silico analysis, in which (1) the nucleotide sequence divergence of 6.5–9.8% was observed between clade A and clade D strains (similar to that between other clades) (Table 2); (2) the strains in clade D formed a distinct cluster from clade A strains supported by a high bootstrap value of 97% (Figure 1A); and (3) amino acid residues in several positions of the VP1 of clade D strains were different from those of clade A strains (Table 3). Intriguingly, two distinct clusters were noted in clade D (Figure 1A), with nucleotide divergence of 4.4–5.9% between strains of the two clusters (Table 2). Moreover, amino acid residues in positions 5, 6, 103, and 279 of the VP1 were different between strains of these two clusters (Table 3). These findings suggested that these two clusters should be classified as subclade D1 and D2 (Figure 1A). Thus, the “A2” strain (case 4) in this study should also belong to subclade D1 (Figure 1A). This and other subclade D1 strains had a high degree of VP1 nucleotide sequence identity of 97.5–99.7% (Table 2).

### 2.3. Partial 5′UTR and 2C Sequence Analysis

Although the VP1 of the strain V14-8157864 from the fatal case (case 1) could not be amplified, the partial 5′UTR and 2C gene region of this strain and the other nine EV-D68 strains from Hong Kong were all successfully amplified and sequenced. Phylogenetic analysis using the partial 5′UTR and 2C sequences (Figure 1B,C) showed similar topologies to those using VP1 sequences. The strain V14-8157864 from the fatal case was clustered with B3 strains.

### 2.4. Complete Genome Analysis of Local and Regional EV-D68 Strains

**Figure 2:**
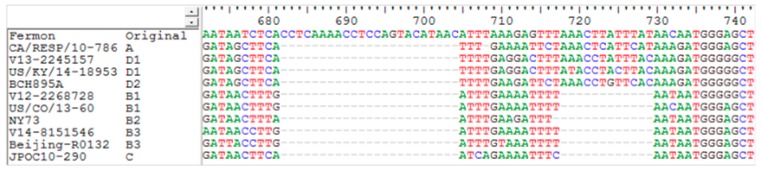
Deletion blocks in 5′UTR of EV-D68 strains. Nucleotides 672–742 (based on the prototype strain with GenBank accession no. AY426531) of the 5′UTR are shown. EV-D68 representative strains of clade A, B, C, D, and the prototype strain Fermon are included.

To look for potential mutation and recombination events among EV-D68 strains in Hong Kong, one strain belonging to each subclade (B1, B3, and D1) were selected for complete genome sequencing (Table 1). The three genomes are around 7.3 kbp in length, after excluding the polyadenylated tract, and the G + C content is 42%. Two deletions (CCTCAAAACCTCCAGTACATAAC and AAACTTATTTAT corresponding to positions 682–704 and 718–729 of the prototype strain Fermon, respectively) in the spacer region of 5′UTR between the end of the internal ribosome entry site and the start codon of the open reading frame were noted in strains belonging to clades B and C when compared to the prototype strain Fermon [23], while only the first deletion (CCTCAAAACCTCCAGTACATAAC) was observed in strains belonging to clades A and D (Figure 2). 

**Figure 3:**
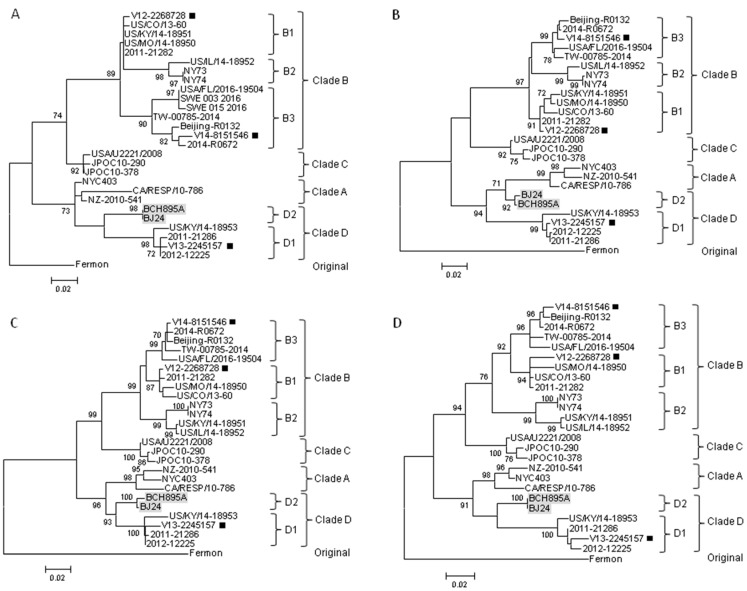

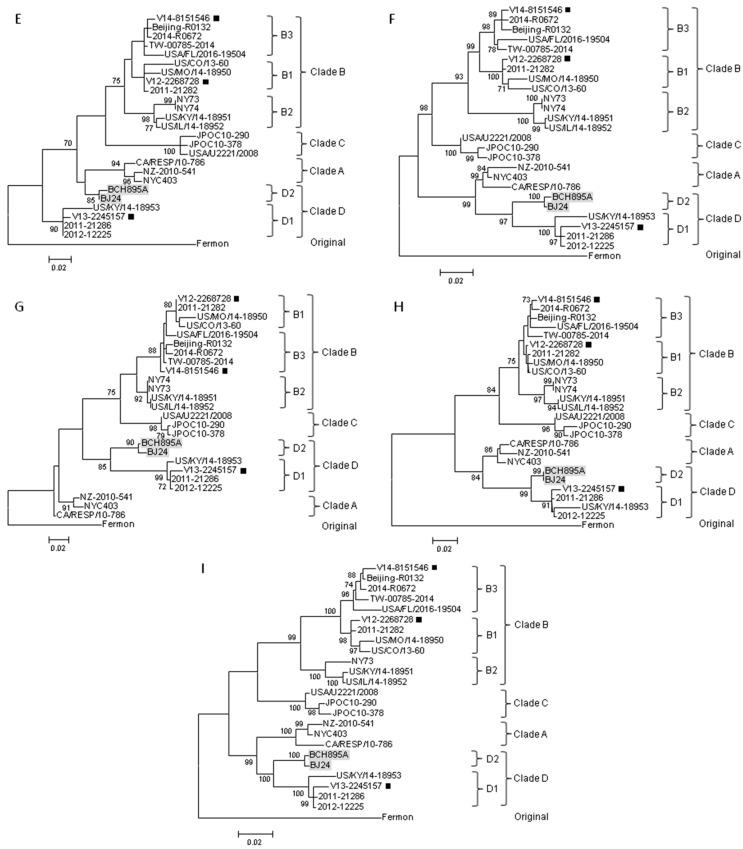
Phylogenetic analysis using various gene sequences of EV-D68 strains. The trees were constructed using the ML method, with bootstrap values calculated from 1000 trees. Sequences for 207 nucleotide positions in each VP4 region (**A**), 744 nucleotide positions in each VP2 region (**B**), 705 nucleotide positions in each VP3 region (**C**), 441 nucleotide positions in each 2A region (**D**), 297 nucleotide positions in each 2B region (**E**), 990 nucleotide positions in each 2C region (**F**), 333 nucleotide positions in each 3A–3B region (**G**), 549 nucleotide positions in each 3C region (**H**) and 1371 nucleotide positions in each 3D region (**I**) were included in the analysis. Bootstrap values expressed as percentages are shown at the nodes and the scale reflects the number of nucleotide substitutions per site along the branches. Only bootstrap values >70% are shown. Black squares indicate the three EV-D68 strains subjected to complete genome sequencing in the present study. The interclade recombinants from mainland China are highlighted in grey.

To examine for incongruent tree topologies which may suggest potential recombination events, phylogenetic trees using nucleotide sequences of VP4 to VP1, 2A to 2C, and 3A to 3D regions of three EV-D68 strains from Hong Kong and other EV-D68 strains with complete genome sequences available in the GenBank were constructed (Figure 1A and Figure 3). The results did not reveal potential recombination in the three Hong Kong strains, and confirmed that V13-2245157 belonged to clade D1, while V12-2268728 and V14-8151546 belonged to clades B1 and B3, respectively (Figure 1A and Figure 3). A study showed that the majority of the outbreak strains belonging to subclade B1 in the US in 2014 differed from others, with signature mutations M291T, V341A, T860N, D927N, S1108G, and R2005K [13,23,24], which were also observed in the isolate V12-2268728 in Hong Kong. The genome sequence of this isolate shared 98% nucleotide identity to those of outbreak strains in the U.S. (subclade B1), supporting the idea that the EV-D68 epidemic in the US may have originated from strains circulating in Asia a few years ago [14].

**Figure 4:**
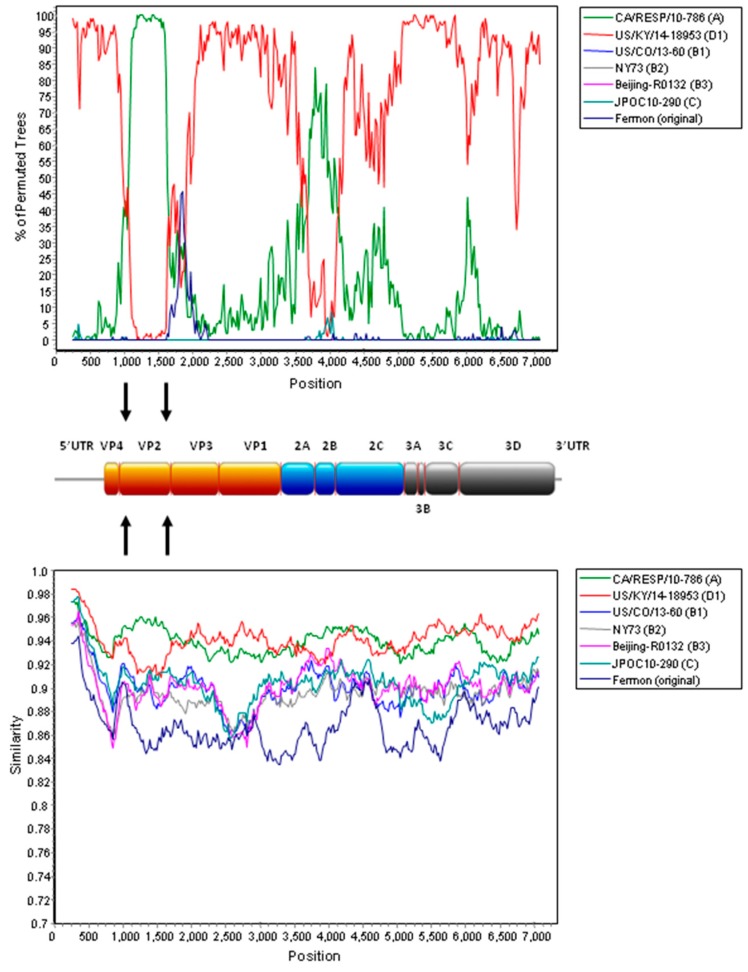
Recombination analysis of the complete genome of EV-D68. Bootscan analysis (**upper** panel) and similarity plot (**lower** panel) were conducted with SimPlot version 3.5.1 (Kimura distance model; window size, 500 bp; step, 20 bp) on a gapless nucleotide alignment, generated with Clustal X version 2.0 (University College Dublin, Dublin, Ireland), with the genome sequence of the EV-D68 strain BCH895A as a query sequence. The red line denotes EV-D68 subclade D1 strain US/KY/14-18953 and the green line denotes EV-D68 clade A strain CA/RESP/10-786. Arrows indicate the gene regions in which potential recombination breakpoints are located.

On the other hand, potential recombination events were identified in two EV-D68 subclade D2 strains from mainland China, as retrieved from the GenBank search, during our analysis. Examination of the tree topologies of the other EV-D68 strains revealed that the EV-D68 subclade D2 strains BCH895A and BJ24 from mainland China were most closely related to the strains of clade A for VP2 and 2B, while they were most closely related to subclade D1 strains for other gene regions (Figure 1A and Figure 3), indicating that recombination events might have occurred in the two subclade D2 strains. Similarity plot and bootscan analyses, using the BCH895A strain as a query sequence, were performed to identify potential recombination sites (Figure 4). The analyses demonstrated that four recombination sites were probably located at VP4–VP2 (between nucleotide positions 900 and 1300), VP2–VP3 (between nucleotide positions 1500 and 1800), 2A–2B (between nucleotide positions 3500 and 3800), and 2B–2C (between positions 4000 and 4300). The region between the VP4–VP2 and VP2–VP3 junctions and the region between the 2A–2B and 2B–2C junctions of the D2 strain were highly similar to the clade A strain, while other regions were most closely related to the D1 strain. Potential recombination within the genome sequences was also examined by the Recombination Detection Program (RDP4). Recombination events were detected in the genome sequence of the strains BCH895A and BJ24, supported by *p* values of less than 0.05 in the GENECONV, Bootscan, and SiScan methods. Two significant recombination breakpoints were identified in the VP2 gene at nucleotide positions 1297 and 1506. While the similarity plot and bootscan analysis supported the recombination event in the 2B region, this lacked statistical support by RDP4. The above findings suggested that the two EV-D68 D2 strains BCH895A and BJ24 identified in mainland China in 2008 represent interclade recombinants with the VP2 acquired from clade A.

## 3. Discussion

This study reported the first fatal case associated with EV-D68 infection in Hong Kong. The 10-year-old boy (case 1) presented with acute encephalitis and deteriorated rapidly after admission. Although co-infection by *M. pneumoniae* might have contributed to the encephalitis, the role of EV-D68 in his illness or exacerbating the mycoplasma infection cannot be excluded. Evidence of neurological complications associated with EV-D68 infection is increasing in recent epidemiological investigations [25,26,27], and the absence of EV-D68 in CSF in the present case may be due to delayed specimen collection and was consistent with previous reports [26,27]. The failure to detect EV-D68 in CSF is not surprising as other known neurotropic enteroviruses, like EV-A71 and poliovirus, were not always detected from CSF specimens of patients with neurological complications, while other specimen types such as throat swabs, rectal swabs, or stool specimens gave higher diagnostic yields [13,28,29,30,31]. Another possible reason is that the neurological symptoms are caused by an aberrant immune response to EV-D68 infection [13]. On the other hand, the detection of EV-D68 in CSF from patients with neurological complications has been reported in two cases [32,33], suggesting the possibility of direct viral neuroinvasion. The latest study demonstrated that EV-D68 strains from the US 2014 outbreak could cause motor neuron death and produce paralytic disease in mice resembling human acute flaccid myelitis (AFM), supporting the causative role of EV-D68 in neurological diseases [34]. Further investigation is therefore needed to delineate whether an indirect immune effect and/or direct damage to the neuron is the key mechanism of causing neurological symptoms by EV-D68 infection. Nevertheless, the two pathogenic mechanisms may not contradict each other, as both processes may co-exist to cause pathologies, as in many other pathogens. Recent studies have shown that neurological symptoms were associated with EV-D68 subclade B3 strains. An outbreak of EV-D68 subclade B3 mainly involving children was reported in Sweden, in which some patients had severe respiratory or neurological symptoms and one died [35]. In another study describing an upsurge of EV-D68 in the Netherlands, some infected children required intensive care unit admission due to respiratory insufficiency and one had AFM that were associated with EV-D68 subclade B3 strains [36]. In Italy, an AFM case that was probably caused by the EV-D68 B3 strain was also reported recently [37]. Unfortunately, the VP1 gene sequence of the EV-D68 strain (V14-8157864) from the present fatal case could not be amplified probably due to low viral load. We have attempted to propagate this strain in cell culture, but were unsuccessful. Yet, the partial 5′UTR and 2C sequences of this strain were 100% identical to those of the subclade B3 strain V14-8151546 (case 10). Interestingly, these two strains, together with the strains identified in the other five young children (cases 5–9), were detected during the summer of 2014, suggesting that similar B3 strains may have been predominating in our population. This is in line with our previous study showing that EV-D68 subclade B3 was the predominant lineage circulating in Hong Kong since 2014 [14]. In fact, subclade B3 strains were also detected in Taiwan in 2014 [22]. It is likely that the recent outbreaks of EV-D68 due to subclade B3 in Europe may have originated from strains that emerged in Asia in 2014. Clinicians should be aware of the possible neurological manifestations of EV-D68 infection, which may help better understand the pathogenicity of the emerging subclade B3 strains. The six mutations (M291T, V341A, T860N, D927N, S1108G, and R2005K) associated with neurovirulence in the US outbreak strains (subclade B1 strains in 2014) [13,23,24] were noted in the B1 strain, but not in the B3 strain in the present study. Further studies are required to examine the role of these mutations in the pathogenicity caused by EV-D68.

The previously recognized subclade A2 strains should be re-designated as subclade D1 within clade D. Current taxonomy and classification of picornaviruses, including enteroviruses, are based on the capsid region, particular VP1 [38,39,40]. In the present study, we have determined the pairwise nucleotide identities using VP1 gene sequences of EV-D68 strains, with the results showing that the average nucleotide sequence divergences between clades and between subclades were 10.8% (6.5–14.8%) and 4.7% (2.6–7.1%), respectively (Table 2). As the average nucleotide divergence between clade A and “A2” strains was 8.4%, together with the evidence from the VP1 sequence and phylogenetic analyses (Figure 1A and Figure 3), all these findings suggested that the “A2” strains should belong to clade D, which has been proposed by other authors [21,22]. Among the clade D strains, two distinct clusters were shown in the phylogenetic tree of VP1 (Figure 1A) and they had an average nucleotide divergence of 5.1% (Table 2). Furthermore, amino acid differences were noted in the VP1 of the strains between these two clusters (Table 3). Therefore, the strains of clade D should be further divided into subclades D1 and D2. In this study, one subclade D1 strain was detected in the elderly (case 4) with pneumonia, which is in line with our previous study showing that adults/the elderly in Hong Kong were exclusively infected by subclade D1 (“A2”) [14]. Although subclade D1 strains have been circulating globally [22,36,41], the clinical characteristics of patients infected by these strains were not reported in other studies. Further epidemiological investigations on subclade D1 strains are needed to determine if this lineage also has a predilection for infecting adults/the elderly in other countries or continents.

We described the first evidence for interclade recombination in EV-D68. Recombination is a well-known phenomenon in enterovirus evolution and recombination breakpoints have been frequently detected in non-structural regions of enterovirus genomes [18,42,43,44]. This is possibly because the non-structural genes are highly conserved at certain regions among the same enterovirus species when compared to structural (capsid) genes. Nevertheless, natural recombination in the capsid region of polioviruses has also been reported [45,46]. To date, only one study has demonstrated recombination between subclades B1 and B2 in an EV-D68 strain from the US during the 2014 outbreak, with a breakpoint in VP2 [19]. In the present study, recombination in capsid region was identified in two EV-D68 strains (BCH895A and BJ24) belonging to subclade D2 from mainland China in 2008. Both phylogenetic and recombination analyses showed that the nucleotide sequences of the two subclade D2 strains from mainland China were most closely related to those of clade A strains in VP2, but to those of clade D strains in other genomic regions, suggesting that interclade recombination had occurred between clade A and D strains of EV-D68. Such recombination is not unexpected since the capsid regions are sufficiently conserved among different clades of a single enterovirus genotype. Though the association between natural enterovirus recombination and pathogenicity remains unclear, an in vitro study showed that replacing a structural region of a slow-growth EV-A71 strain with that of a fast-growth EV-A71 strain could generate a recombinant virus with growth improvement and larger plaque phenotypes [47]. Further studies are warranted to investigate the role of recombination in driving the evolution of EV-D68.

In conclusion, we described the molecular epidemiology of EV-D68 in Hong Kong and presented further evidence for taxonomic reclassification of subclade A2 into a separate clade D1. We also showed for the first time that recent subclade D2 strains from mainland China probably originated from an interclade recombination event between clade A and clade D strains. Further evolutionary analysis of currently circulating EV-D68 strains in East Asia is warranted. Continuous epidemiological and laboratory surveillance is required to detect novel variants with epidemic potential.

## 4. Materials and Methods

### 4.1. Clinical Specimens

NPAs or NPSs were collected from 10 hospitalized patients from seven regional hospitals in Hong Kong during the years from 2012–2014 (Table 1). EV-D68 detection and isolation from NPAs or NPSs was performed at the Public Health Laboratory Centre, Centre for Health Protection, Department of Health, Hong Kong. Detection of EV-D68 RNA from clinical specimens was performed using real-time RT-PCR targeted to the 5′UTR region and sequencing using a previously described protocol [48]. Isolation of EV-D68 was performed using the human embryonal rhabdomyosarcoma (RD) cell line. The clinical features, laboratory results, and outcome of illness of these patients were analyzed. The ethical approval was obtained from the Institutional Review Board of the University of Hong Kong/Hospital Authority Hong Kong West Cluster (UW 16-365; approval date: 20 July 2016) for this study.

### 4.2. RNA Extraction

The clinical specimens (200 µL each) were subjected to total nucleic acid extraction by the EZ1 Virus Mini Kit v2.0 (QIAGEN, Hilden, Germany), with the elution volume of 60 µL. The eluate was used as a template for reverse-transcription polymerase chain reaction (RT-PCR).

### 4.3. Reverse-Transcription Polymerase Chain Reaction (RT-PCR) and Sequencing of VP1 of EV-D68 for Clade Determination

RT was performed using random hexamers and a SuperScript III Kit (Invitrogen, Carlsbad, CA, USA) as described previously [14,18]. The VP1 of EV-D68 strains detected from the clinical specimens were amplified and sequenced using the primers and the strategy described in our previous publication [14]. Both strands of PCR products were sequenced twice with an ABI 3130xl DNA Analyzer (Applied Biosystems, Foster City, CA, USA) using the PCR primers. A phylogenetic tree of the VP1 was constructed using maximum likelihood (ML) method in MEGA6 with the model T92 + I [49], with bootstrap analysis of 1000 replicates. The percentage of VP1 nucleotide sequence identity was determined by MatGAT2.01 software (Montclair State University, Montclair, NJ, USA) [50].

### 4.4. RT-PCR and Sequencing of Partial 5′UTR and 2C of EV-D68 and Phylogenetic Analysis

Due to the unsuccessful amplification of the VP1 of the EV-D68 strain V14-8157864 from the fatal case, we attempted to amplify and sequence the partial 5′UTR and 2C of this strain, together with the EV-D68 strains detected from the other 9 patients using the primers and the strategy described in our previous publication [14]. Phylogenetic trees of each region were constructed using ML method in MEGA6 with the model K2 + G for partial 5′UTR and T92 + I for partial 2C [49], with bootstrap analysis of 1000 replicates.

### 4.5. Complete Genome Sequencing of EV-D68

The complete genomes of three EV-D68 strains (V12-2268728, V13-2245157, and V14-8151546) were amplified and sequenced using the strategy described in our previous publications [18,51]. The cDNA was synthesized using the viral RNA by a combined random-priming and oligo(dT) priming strategy. PCR amplification was achieved by degenerate primers designed by multiple alignments of the EV-D68 genomes and other primers designed based on the results of the first and subsequent rounds of sequencing. The primer sequences are available upon request. The 5′ ends of the EV-D68 genomes were confirmed by rapid amplification of cDNA ends (RACE) using Terminal Deoxynucleotidyl Transferase, recombinant (Invitrogen, Carlsbad, CA, USA). Sequences were assembled and manually edited to produce final sequences of the viral genomes by BioEdit version 7.2.5 (NC State University, Raleigh, NC, USA).

### 4.6. Genome Analysis

The nucleotide sequences of various genomic regions of the EV-D68 strains detected in Hong Kong were compared to those of EV-D68 strains with available genome sequences in GenBank (Table A1). Phylogenetic tree construction was performed using the ML method in MEGA6 with the models T92 + G for VP4, VP2, 2B, 3A-3B, and 3C, and T92 + I for VP3, VP1, 2A, 2C, and 3D (Figure 1A and Figure 3), with bootstrap values calculated from 1000 trees [49]. Recombination analysis was conducted using a nucleotide alignment of the genome sequences of the EV-D68 strains in this study, EV-D68 prototype strain Fermon (original), strain CA/RESP/10-786 (clade A), strain US/KY/14-18953 (clade D1), strain US/CO/13-60 (clade B1), strain NY73 (clade B2), strain Beijing-R0132 (clade B3), and strain JPOC10-290 (clade C) was generated by ClustalX version 2.0 [52], and edited manually. Once aligned, similarity plot and bootscan analyses were performed using SimPlot version 3.5.1 (National Institute of Virology, Pune, India) (window size, 500 bp; step, 20 bp) [53]. Potential recombination within the genome sequences of EV-D68 was also examined by the Recombination Detection Program version 4.46 (RDP4) (University of Cape Town, Cape Town, South Africa) [54]. Phylogenetic incongruence between different regions and with *p* values of less than 0.05 in the methods further supported the presence of recombination events.

### 4.7. Nucleotide Sequence Accession Numbers

The complete genome sequences of the three EV-D68 strains and the VP1, partial 5′UTR, and 2C sequences of the EV-D68 strains (Table 1) have been deposited in the GenBank database under accession numbers KY767820 to KY767842.

## Figures and Tables

**Table 1:** Clinical characteristics of the 10 cases of enterovirus D68 (EV-D68) infections.

Case	Strain	Specimen Type	Collection Date	Gender	Age	Underlying Diseases	Diagnosis	Clade
1	V14-8157864	NPA	16 August 2014	M	10	α-thalassemia trait	Encephalitis, *Mycoplasma pneumoniae* co-infection	B3 ^#^
2	V12-2240471	NPS	7 May 2012	M	5	Allergic rhinitis	URTI, asthma	B1
3	V12-2268728 *	NPA	20 July 2012	M	2	None	URTI, asthma	B1
4	V13-2245157 *	NPS	21 May 2013	F	94	HT, CVA, gout, dementia	Pneumonia	D1
5	V14-8133616	NPA	18 May 2014	M	4	Allergic rhinitis, asthma	URTI, asthma	B3
6	V14-8135150	NPA	23 May 2014	F	4	MRSA skin infection	URTI, RSV co-infection	B3
7	V14-8143594	NPS	21 June 2014	F	4	Eczema	Wheezy bronchitis	B3
8	V14-8143833	NPS	24 June 2014	F	4	None	URTI, asthma	B3
9	V14-8151518	NPS	23 July 2014	M	5	α-thalassemia trait	URTI, Henoch Schonlein purpura	B3
10	V14-8151546 *	NPS	23 July 2014	M	5	None	Febrile wheeze	B3

* EV-D68 strains selected for complete genome sequencing. ^#^ VP1 sequence not available; partial 5′UTR and 2C were sequenced (100% identical to those of EV-D68 strain V14-8151546; potential subclade B3). Abbreviations: CVA, cerebrovascular accident; HT, hypertension; MRSA, methicillin-resistant *Staphylococcus aureus*; NPA, nasopharyngeal aspirate; NPS, nasopharyngeal swab; RSV, respiratory syncytial virus; URTI, upper respiratory tract infection; M: male; F: female.

**Table 2:** Nucleotide sequence identities of VP1 genes between clades and subclades of EV-D68 strains.

Strains *	Pairwise Identity (%)	Average Nucleotide Divergence (%) [Range]
Interclade		10.8 [6.5–14.8]
Prototype vs. A	87–87.9	
Prototype vs. B	85.5–86.6	
Prototype vs. C	87.8–88	
Prototype vs. D	85.2–86.7	
A vs. B	86.5–90.4	
A vs. C	88.8–91.4	
A vs. D	90.2–93.5	
B vs. C	89.8–93.1	
B vs. D	86.4–89.7	
C vs. D	87.9–90.5	
A vs. D1	90.2–91.7	
A vs. D2	91.8–93.5	
Intersubclade		4.7 [2.6–7.1]
B1 vs. B2	94–95.2	
B1 vs. B3	94.7–97.4	
B2 vs. B3	92.9–94.2	
D1 vs. D2	94.1–95.6	
Intrasubclade		1.4 [0–3.5]
B1	96.5–99.8	
B2	98.9–100	
B3	97.3–99.9	
D1	97.5–99.7	
D2	98.1–99.6	

* VP1 sequences of the EV-D68 strains used for the analysis are shown in Table A1 in Appendix A.

**Table 3:** Amino acid residues of VP1 between EV-D68 strains for clade A and D differentiation.

Strains	Positions												
	2	5	6	92	103	131	140	142	147	177	186	279	C-terminus
Clade A													
CA/RESP/10-786	D	D	A	A	F	V	S	S	T	A	I	R	VTT
NZ-2010-541	D	D	A	A	F	V	S	S	T	A	I	R	VTT
NYC403	D	D	A	A	F	V	S	S	T	A	I	R	VTT
TW-02378_2010	D	D	A	A	F	V	S	S	T	A	I	R	
ITA/19391/12	D	H	A	A	F	V	S	S	T	A	I	R	
HK2/2010	D	D	A	A	F	V	S	S	T	A	I	R	VTT
Clade D													
Subclade D1:													
TW-02722_2013	E	H	E	T	Y	I	G	N	M	G	V	K	RLVNT
US/KY/14-18953	E	H	E	T	Y	I	G	N	M	G	V	K	RLVNT
2011-21286	E	H	E	T	Y	I	G	N	M	G	V	K	RLVNT
2012-12225	E	H	E	T	Y	I	G	N	M	G	V	K	RLVNT
2013-1017-26	E	H	E	T	Y	I	G	N	M	G	V	K	RLVNT
HK5/2011	E	H	E	T	Y	I	G	N	M	G	V	K	RLVNT
HK13/2012	E	H	E	T	Y	I	G	N	M	G	V	K	RLVNT
HK21/2013	E	H	E	T	Y	I	G	N	M	G	V	K	RLVNT
V13-2245157	E	H	E	T	Y	I	G	N	M	G	V	K	RLVNT
Subclade D2:													
BCH895A	E	D	A	T	F	I	G	N	M	G	V	R	RLVNT
BJ24	E	D	A	T	F	I	G	N	M	G	V	R	RLVNT
TW-01166_2007	E	D	A	T	F	I	G	N	M	G	V	R	
TW-11134_2009	E	D	A	T	F	I	G	N	M	G	V	R	
1737-Yamagata-2008	E	D	A	T	F	I	G	N	M	G	V	R	RLVNT

The VP1 amino acid residues of EV-D68 subclade D1 different from those of subclade D2 are highlighted in grey. Amino acid code: D, aspartic acid; A, alanine; F, phenylalanine; V, valine; S, serine; T, threonine; I, isoleucine; R, arginine; E, glutamic acid; H, histidine; Y, tyrosine; G, glycine; N, asparagine; M, methionine; K, lysine; L, leucine.

## Abbreviations

AFM	Acute flaccid myelitis
CVA	Cerebrovascular accident
CSF	Cerebrospinal fluid
EV-D	Enterovirus species D
HT	Hypertension
kbp	Kilobase pairs
ML	Maximum likelihood
MRMP	Macrolide-resistant *Mycoplasma pneumoniae*
MRSA	Methicillin-resistant *Staphylococcus aureus*
NPA	Nasopharyngeal aspirate
NPS	Nasopharyngeal swab
RACE	Rapid amplification of cDNA ends
RD	Rhabdomyosarcoma
RDP	Recombination Detection Program
RSV	Respiratory syncytial virus
RT-PCR	Reverse transcription polymerase chain reaction
URTI	Upper respiratory tract infection
US	United States
UTR	Untranslated region

## Appendix A

**Table A1:** List of EV-D68 strains used for analysis in the present study.

Strain	Year of Isolation	Place of Isolation	Clade *	Source	GenBank Accession No.
Fermon (prototype)	1962	USA	Original	GenBank	AY426531
CA/RESP/10-786	2013	USA	A	GenBank	KM892500
NYC403	2009	USA	A	GenBank	JX101846
NZ-2010-541	2010	NZ	A	GenBank	JX070222
HK2/2010	2010	HK	A	GenBank	KT959174 (VP1),
					KT959114 (2C)
TW-02378_2010	2010	TW	A	GenBank	KP657713 (VP1)
ITA/19391/12	2012	Italy	A	GenBank	KC763159 (VP1)
2011-21282	2011	China	B1	GenBank	KT285320
HK8/2011	2011	HK	B1	GenBank	KT959180 (VP1),
					KT959120 (2C)
HK14/2012	2012	HK	B1	GenBank	KT959186 (VP1)
					KT959126 (2C)
HK26/2014	2014	HK	B1	GenBank	KT959198 (VP1),
					KT959138 (2C)
NY316	2014	USA	B1	GenBank	KP745764
STL_2014_12	2014	USA	B1	GenBank	KM881710
US/CO/13-60	2013	USA	B1	GenBank	KP100794
US/CO/14-93	2014	USA	B1	GenBank	KP126911
US/MO/14-18950	2014	USA	B1	GenBank	KM851228
NY73	2014	USA	B2	GenBank	KP745768
NY74	2014	USA	B2	GenBank	KP745769
US/KY/14-18951	2014	USA	B2	GenBank	KM851229
US/IL/14-18952	2014	USA	B2	GenBank	KM851230
2014-R0672	2014	China	B3	GenBank	KT280500
Beijing-R0132	2014	China	B3	GenBank	KP240936
HK25/2014	2014	HK	B3	GenBank	KT959197 (VP1),
					KT959137 (2C)
HK28/2014	2014	HK	B3	GenBank	KT959200 (VP1),
					KT959140 (2C)
TW-00932-2014	2014	TW	B3	GenBank	KT711081
TW-00785-2014	2014	TW	B3	GenBank	KT711083
NY10_16	2016	USA	B3	GenBank	KX957754
USA/FL/2016-19504	2016	USA	B3	GenBank	KX675261
SWE_003_2016	2016	SWE	B3	GenBank	KY215829 (VP4)
SWE_015_2016	2016	SWE	B3	GenBank	KY215841 (VP4)
USA/U2221/2008	2008	USA	C	GenBank	KX255371
USA/MO93/2009	2009	USA	C	GenBank	KX261814
JPOC10-290	2010	Japan	C	GenBank	AB601882
JPOC10-378	2010	Japan	C	GenBank	AB601883
2011-21286	2011	China	D1	GenBank	KT306743
2012-12225	2012	China	D1	GenBank	KT285319
2013-1017-26	2013	China	D1	GenBank	KT280501
HK5/2011	2011	HK	D1	GenBank	KT959177 (VP1),
					KT959117 (2C)
HK13/2012	2012	HK	D1	GenBank	KT959185 (VP1),
					KT959125 (2C)
HK21/2013	2013	HK	D1	GenBank	KT959193 (VP1),
					KT959133 (2C)
US/KY/14-18953	2014	USA	D1	GenBank	KM851231
TW-02722_2013	2013	TW	D1	GenBank	KP657720 (VP1)
BCH895A	2008	China	D2	GenBank	KF726085
BJ24	2008	China	D2	GenBank	KU242683
1737-Yamagata-2008	2008	Japan	D2	GenBank	AB667899 (VP1)
TW-01166_2007	2007	TW	D2	GenBank	KP657701 (VP1)
TW-11134_2009	2009	TW	D2	GenBank	KP657708 (VP1)
V12-2240471	2012	HK	B1	This study	KY767823 (VP1),
					KY767829 (5′UTR),
					KY767836 (2C)
V12-2268728	2012	HK	B1	This study	KY767820
V13-2245157	2013	HK	D1	This study	KY767821
V14-8133616	2014	HK	B3	This study	KY767824 (VP1),
					KY767830 (5′UTR),
					KY767837 (2C)
V14-8135150	2014	HK	B3	This study	KY767825 (VP1),
					KY767831 (5′UTR),
					KY767838 (2C)
V14-8143594	2014	HK	B3	This study	KY767826 (VP1),
					KY767832 (5′UTR),
					KY767839 (2C)
V14-8143833	2014	HK	B3	This study	KY767827 (VP1),
					KY767833 (5′UTR),
					KY767840 (2C)
V14-8151518	2014	HK	B3	This study	KY767828 (VP1),
					KY767834 (5′UTR),
					KY767841 (2C)
V14-8151546	2014	HK	B3	This study	KY767822
V14-8157864	2014	HK	B3 ^#^	This study	KY767835 (5′UTR),
					KY767842 (2C)

^#^ VP1 sequence not available; partial 5’UTR and 2C were sequenced (100% identical to those of EV-D68 strain V14-8151546; potential subclade B3). Abbreviations: HK, Hong Kong; NZ, New Zealand; SWE, Sweden; TW, Taiwan; USA, the United States of America.
